# Dietary betaine activates hepatic *VTGII* expression in laying hens associated with hypomethylation of *GR* gene promoter and enhanced *GR* expression

**DOI:** 10.1186/s40104-017-0218-9

**Published:** 2018-01-18

**Authors:** Nagmeldin A. Omer, Yun Hu, Yan Hu, Abdulrahman A. Idriss, Halima Abobaker, Zhen Hou, Haibo Dong, Ruqian Zhao

**Affiliations:** 1 MOE Joint International Research Laboratory of Animal Health & Food Safety, Nanjing Agricultural University, Nanjing, 210095 People’s Republic of China; 2 Key Laboratory of Animal Physiology & Biochemistry, Nanjing Agricultural University, Nanjing, 210095 People’s Republic of China; 3 Jiangsu Collaborative Innovation Centre of Meat Production and Processing, Quality and Safety Control, Nanjing, 210095 People’s Republic of China; 4 Poultry Institute, Chinese Academy of Agriculture Sciences, Yangzhou, Jiangsu China

**Keywords:** Betaine, Glucocorticoid receptor, Hypomethylation, Laying hens, Vitellogenin

## Abstract

**Background:**

Vitellogenin (VTG) is a precursor of egg yolk proteins synthesized within the liver of oviparous vertebrates. Betaine is an important methyl donor that is reported to improve egg production of laying hens with an unclear mechanism. In the present study, we fed betaine-supplemented diet (0.5%) to laying hens for 4 wk and investigated its effect on *VTGII* expression in the liver.

**Results:**

Betaine did not affect chicken weight, but significantly (*P* < 0.05) increased egg laying rate accompanied with a significant (*P* < 0.05) increase in hepatic concentration and plasma level of VTGII. Plasma estrogen level did not change, but the hepatic expression of estrogen receptor α (*ERα*) mRNA was significantly (*P* < 0.05) up-regulated. Betaine did not affect the protein content of ERα, but significantly (*P* < 0.05) increased hepatic expression of glucocorticoid receptor (*GR*) at both mRNA and protein levels. Also, ERα/GR interaction tended to be enhanced in the liver nuclear lysates of betaine-supplemented hens as determined by co-immunoprecipitation. Furthermore, dietary betaine supplementation significantly increased (*P* < 0.05) the hepatic expression of methyl-transfer enzymes, such as BHMT, GNMT, and DNMT1, which was associated with higher SAM/SAH ratio and hypomethylation of *GR* promoter regions.

**Conclusions:**

Betaine activates hepatic *VTGII* expression in association with modified DNA methylation of *GR* gene promoter, *GR* expression and ERα/GR interaction. Activation of hepatic *VTGII* expression may contribute, at least partly, to improved egg production in betaine-supplemented hens.

## Background

Vitellogenin (VTG) is one of the major yolk proteins expressed exclusively in the liver of mature females in oviparous vertebrates [1]. After hepatic synthesis, VTG is secreted into the bloodstream and selectively incorporated into the growing oocytes, where it is cleaved to make other yolk proteins [2, 3]. It is lipoglycophosphoprotein synthesized during the period of reproduction and stored as an essential nutritional reservoir for the developing embryo [3]. Three subtypes of VTGs have been identified in the chicken, among which *VTGII* is the most abundant [4]. Sekimoto et al. found a close relationship between decreased egg production and lower VTG concentration in laying hens subjected to food and water deprivation [5]. Liou et al. reported a positive correlation between serum level of VTG and total egg production in Taiwan red feathered chickens [6]. These findings indicate a positive contribution of hepatic VTG synthesis to egg laying performance.

Hepatic *VTG* expression in birds has long been used as a model for studying the mechanism of hormone action [7, 8]. The transcriptional regulation of *VTG* in vertebrates is directly under the control of female sex hormones. Estrogen (E2) is used for the induction of *VTG* expression. E2 action is mediated through its nuclear receptor (ER) that binds to the estrogen responsive element (ERE) located in the regulatory region of estrogen-responsive genes [9]. Two isoforms of ER have been identified (α and β). ERα represents the most predominant form [10] that plays a major role in the transactivation of chicken *VTGs* [4, 11]. In addition to E2, several other hormones have been reported to synergize *VTG* production, among which are prolactin and growth hormone in fish hepatocytes [12, 13], and growth hormone in reptiles and frogs [14–16]. On the contrary, testosterone and progesterone are found to reduce estrogen-induced *VTG* mRNA expression in male turtles [17]. Glucocorticoid receptor (GR) regulates gene transcription through binding to the glucocorticoid response element (GRE) in the promoter of its target genes. Moreover, GR is able to modulate the activity of other transcription factors through protein-protein interactions [18]. Various studies revealed ER/GR interaction in *ER*(+) breast cancer cells. For instance, West et al. found an association of ER with GREs in breast cancer cells [19], while Karmakar et al. demonstrated GR binding to several EREs [20]. Chicken *VTGII* gene is subjected to multihormonal regulation by some steroid hormones including glucocorticoids and estrogen [21]. A GRE was found to overlap with an ERE within a region of *VTGII* promoter [22]. However, the contribution of GR in transcriptional regulation of *VTGII* is not yet studied.

Betaine, also referred to as trimethylglycine, is a naturally occurring nutrient that was first discovered in sugar beets and was later found in several micro-organisms, marine invertebrates, plants and animals [23]. Many studies indicate that betaine exerts significant nutritional and physiological functions [24], such as growth promotion, anti-stress, reproductive performance improvement, antioxidation, as well as osmotic protection [25]. Studies on swine and poultry have suggested that betaine supplementation can decrease overall fat deposition, and improve carcass characteristics by stimulating lipolysis [26, 27]. Different studies indicate that feeding betaine to laying hens has a positive effect on laying performance and egg production [28–30]. Nevertheless, the mechanism behind these beneficial effects remains largely unknown. Furthermore, no data has been published to connect the impact of betaine on egg production with hepatic expression of *VTG*.

Betaine is an important methyl donor in the one-carbon metabolic pathway, providing methyl group for the conversion of homocysteine to methionine in a reaction catalyzed by betaine homocysteine methyltransferase (BHMT) [31]. Methionine is then converted to S-adenosylmethionine (SAM) [32]. SAM acts as a universal methyl donor for many methylation processes, including DNMTs-catalyzed DNA methylation that are essential for the epigenetic regulation of gene expression [33]. DNA cytosine-guanine dinucleotides (CpGs) methylation in the promoter regions is generally involved in the blocking of promoter accessibility and therefore gene silencing. Conversely, demethylation of gene promoter sequences results in gene activation [34]. The promoter regions of *GR* gene are rich in CpGs which are susceptible to DNA methylation [35]. CpG methylation of *GR* promoter greatly affects its transcription [36]. Recently, we reported that maternal betaine supplementation modulates *GR* expression in neonatal piglets through modification of DNA methylation [37, 38]. However, studies are lacking concerning the effect of betaine on *GR* promoter methylation and expression in the chicken.

Therefore, the aim of the current study was to investigate the effect of dietary betaine supplementation on hepatic expression of *VTGII* in laying hens. Moreover, we examined the methylation status of *GR* gene promoter, in association with *GR* expression and GR/ERα interaction, in order to elucidate the possible mechanisms underlying the betaine action.

## Methods

### Experimental design

One hundred and twenty Rugao yellow breeder laying hens (267 d of age) were randomly divided into two groups (60 in each group). Hens in the betaine (BET) group were fed diet containing 0.5% betaine (75% purity; SKYSTONE FEED CO., LTD, Jiangsu, China) for 4 wk, while those in the control (CON) group fed basal diet (Table 1). The laying performance was recorded daily throughout the experimental period. At the end of the experiment, twelve hens were randomly selected from each group, weighed and killed by rapid decapitation. Blood samples were taken, and plasma was separated and stored at −20 °C. Liver samples were dissected and snap frozen in liquid nitrogen and stored at −80 °C.

**Table 1:** Composition of the experimental diets

Ingredient, %	Control	Betaine
Corn	65.00	65.00
Soybean meal	24.67	24.67
Shell powder	6.70	6.70
Limestone	2.03	2.03
Salt	0.30	0.30
Dicalcium phosphate	0.83	0.83
Zeolite	0.01	0.01
Choline chloride	0.17	0.17
Methionine	0.12	0.12
Vitamin premix^a^	0.03	0.03
Minerals premix^b^	0.10	0.10
Betaine	0	0.5

^a^The vitamins premix contain (per kg): vitamin D_3_: 9,000,000 IU; vitamin K: 35,000,000 IU; vitamin B_1_: 10 g; vitamin B_2_: 28 g; vitamin B_6_: 12 g; vitamin B_12_: 80 mg; vitamin E: 140 g; vitamin K_3_: 9 g; *D*-biotin: 5.60 g; *D*-pantothenic acid: 36 g; folic acid: 3.5 g; niacinamide: 100 g; ethoxyquin:1.65 g

^b^The minerals premix contain (per kg): Cu: 6.4 g; Fe: 72 g; Zn: 64 g; Mn: 72 g; Se: 240 mg; I: 480 mg

### Measurement of plasma and hepatic concentration of vitellogenin

Plasma VTG concentration was measured using an Enzyme-Linked Immunosorbent Assay kit (NO. MM-065801, Jiangsu Green Leaf Biotechnology Co. Ltd., China). The assay range of the kit was between 20 ng/mL and 640 ng/mL. The intra- and inter-assay coefficients of variations were less than 10% and less than 15%, respectively. Hepatic VTG concentration was measured from tissue homogenate of phosphate buffer saline (PBS) by using the same kit.

### Measurement of plasma estradiol concentration

Plasma E2 concentration was tested using a radioimmunoassay kit (NO. B05PZB) purchased from Beijing North Institute of Biological Technology Co. Ltd., China. The sensitivity of this kit was less than 2 pg/mL. The intra- and inter-assay coefficients of variations were less than 10% and less than 15%, respectively. The cross-reactivity with estriol, progesterone and testosterone were 0.016%, 0.01% and 0.01%, respectively.

### Total RNA isolation and real-time PCR

Total RNA was isolated from 40 mg liver samples using 1 mL of TRIzol reagent (Invitrogen, USA) according to the manufacturer’s instruction. One microgram of total RNA was reverse-transcribed to cDNA using HiScript QRT SuperMix (Vazyme Biotech, Nanjing, China). Two microliters of diluted cDNA (1:50, vol/vol) was used for real-time PCR with QuantStudio® 6 Flex Real-Time PCR System (Applied Biosystems, USA). PCR conditions were set as follows: initial denaturation at 95 °C for 5 min, followed by 40 cycles of denaturation at 95 °C for 10 s and annealing-extension at 60 °C for 30 s. To confirm the absence of genomic DNA contamination in the RNA preparations, RNA extracts were treated with DNase before reverse transcription, then DNase-treated RNA samples were directly used as a template for PCR to ensure that there was no specific amplification. *β-actin* was used as an internal control to normalize the technical variations. Primers for qRT-PCR (Table 2) were synthesized by Genewiz (Suzhou, China). Data were analyzed using the method of 2^-ΔΔCT^ [39].

**Table 2:** Nucleotide sequences of specific primers

Target genes	GenBank accession	Primer sequences (5′ to 3′)	PCR products, bp
Primers used for QRT-PCR:			
*BHMT*	XM_414685.3	F: TCTTCCTGAATTTCCCTT R: TGAACATCCCATCTAGTGA	57
*GNMT*	XM_015283546.1	F: GGAGGAGGGCTTCCAAGTGA R: GCTCCAGCGTCAGCCAGTT	140
*DNMT1*	NM_206952.1	F: CGAGTGGGACGGCTTCTT R: AGGCGATAGGTGTCAGGGA	144
*VTGII*	NM_001031276.1	F: GAATCCTTCTCGACAAGCCAG R: GCTTCAGCAAAGACGTTCCAG	186
*ERα*	NM_205183.2	F: TAGTTCCGCTCTACGACCTCTT R: AGTTGGTTTCGGTTCTCCTCTT	106
*ERβ*	NM_204794.2	F: AGCGTGTTATGGTCTGCTCC R: GCTCTTAGGCTGCTCTGCAT	87
*GR*	NM_001037826.1	F: CTTCCATCCGCCCTTCA R: TCGCATCTGTTTCACCC	203
*β-actin*	NM_205518.1	F: ATG GCTCCGGTATGTGCA A R: TGTCTTTCTGGCCCATACCAA	120
Primers used for Bisulfite sequencing of *GR* promoter:			
Segment 1		F: TGTTATTTTGTAGAAGTGGGTGTGTTAG R: TAAACAACTTCRCACAACCCATTC	
Segment 2		F: GAATGGGTTGTGYGAAGTTGTTTA R: TTAAAATAACCCRACCRAATACTCC	
Segment 3		F: GTAYGGAGTYGTTTTGTTGGTTG R: ACTATCCTCCAAACCCATCTAATACC	
Segment 4		F: TTATTTGTAAATGTGGGGTTTATTTATAG R: TATAAACACTCCCCAACTCTACTATAACC	

### Protein extraction and western blotting

Total protein was extracted from 40 mg frozen liver sample as previously described [40]. Nuclear protein was extracted from 60 mg liver sample using a special kit (NO. P0028; Beyotime Biotechnology, Wuhan, China) according to the manufacturer’s instruction. The cytoplasmic and nuclear components were then subjected to Western blotting. Histone H1 (BS1655, Bioworld, USA, diluted 1:500) and β-tubulin (AP0064, Bioworld, USA, diluted 1:5,000) were used as markers for nuclear and cytoplasm proteins, respectively. Protein concentrations were measured with a Pierce BCA Protein Assay kit (NO. 23225; Thermo Scientific, USA). Western blot analysis for BHMT (15965–1-AP, Proteintech, USA, diluted 1:500), glycine N-methyltransferase (GNMT) (18,790, Proteintech, USA, diluted 1:500), DNA methyltransferases 1 (DNMT1) (24206–1-AP, Proteintech,USA, diluted 1:500), estrogen receptor alpha (ERα) (MA5–13065, Thermo Fisher, USA, diluted 1:500), and glucocorticoid receptor (GR) (4161, Cell signaling Technology, USA, diluted 1:500) was carried out according to the recommended protocols provided by the manufacturers. β-actin (AP0060, Bioworld, USA, diluted 1:10,000) was used as loading control. All antibodies in the present study were selected based on previous publications in chickens, or when the chicken is stated as tested organism in the product data sheets. Images were captured by VersaDoc 4000MP system (Bio-Rad, USA) and the band density was analyzed with Quantity One software (Bio-Rad, USA).

### Bisulfite sequencing analysis of *GR* promoter CpG methylation

Liver genomic DNA was isolated by phenol/chloroform method. Bisulfite Amplicon Sequencing (BSAS) was used for quantitative methylation analysis. Bisulfite conversion of 1 μg genomic DNA was performed with the EZ DNA Methylation™-GOLD Kit (ZYMO RESEARCH, CA, USA) according to the manufacturer’s protocol. Sodium bisulfite preferentially deaminates unmethylated cytosine residues to thymines, whereas methyl-cytosines remain unmodified. After PCR amplification (HotStarTaq polymerase kit, TAKARA, Tokyo, Japan) of target CpG regions and library construction, the products were sequenced on Illumina MiSeq Benchtop Sequencer (CA, USA). Four segments of CpG islands of GR gene promoter were selected and sequenced. Primers used in the PCR are listed in Table 2. All samples achieved a mean coverage of >600×. Each tested CpG site was named as its relative distance (in bp) to translation start codon (ATG). Methylation level at each CpG site was calculated as the percentage of the methylated cytosines over the total tested cytosines. The average methylation level was calculated using methylation levels of all measured CpG sites within the promoter segment.

### ERα/GR co-immunoprecipitation

Co-immunoprecipitation was performed as previously described with minor modifications [41]. In brief, 500 μg of total protein were incubated with 20 μL of 50% protein A/G plus beads for 2 h at 4 °C, then centrifuged at 7,500×g for 1 min. The supernatants were incubated with 4 μg ERα antibody and rotated overnight at 4 °C. Thereafter, 20 μL of agarose beads were incubated with the protein-antibody complexes for 10 h at 4 °C. After centrifugation, the agarose beads were washed with cold PBS and the immunoprecipitated proteins were run on 10% SDS-polyacrylamide gel for Western blot analysis.

### Statistical analysis

All data are presented as means ± SEM. The differences between groups were analyzed using independent-samples *t*-test with SPSS18.0 for Windows, and were considered statistically significant when *P* < 0.05. For bisulfite sequencing analysis, Chi-square test was used to examine the differences in the methylation level at each CpG site, whereas *t*-test was used to analyze the differences in the average methylation status of each *GR* promoter region. *P* < 0.05 was considered significant.

## Results

### Body weight, liver weight and egg laying rate.

Dietary betaine supplementation significantly increased the egg-laying rate (*P <* 0.05) without significant change in egg weight (Table 3). Hens fed betaine-supplemented diet had similar body weight but heavier liver weight (*P <* 0.01), when compared to their control counterparts.

**Table 3:** Body weight, liver weight, liver index and laying rate

Parameters	CON (*n* = 11)	BET (*n* = 11)	*P* value
Body weight, kg	1.39 ± 0.01	1.40 ± 0.01	0.60
Liver weight, g	25.7 ± 0.9	29.5 ± 1.0	0.02
Liver index, %	1. 9 ± 0.1	2.1 ± 0.1	0.02
Average daily laying rate, %^a^	80.2 ± 1.1	84.5 ± 0.1	0.03
Egg weight, g^a^	43.1 ± 0.4	42.0 ± 0.4	0.31

Values are means ± SEM

^a^Egg laying rate and Egg weight were calculated from the egg laid by 60 chickens in each group

### Hepatic expression and plasma concentration of VTG

**Fig. 1:**
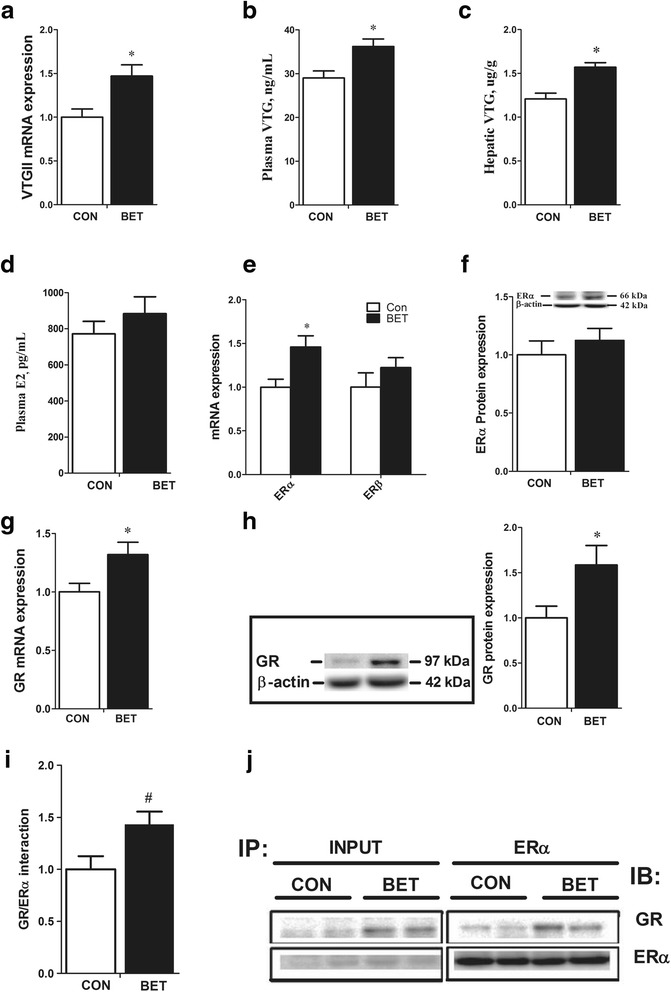
Expression of *VTG, ERα, GR* and co-immunoprecipitation of ERα/GR. **a**) Hepatic expression of *VTGII* mRNA; **b**) Plasma concentration of VTG; **c**) Hepatic concentration of VTG protein; **d**) Plasma E2 concentration; **e**) hepatic mRNA expression of *ERα* and *ERβ*; **f**) Hepatic protein expression of ERα; **g**) Hepatic *GR* mRNA expression; **h**) GR protein expression; **j**) Immuno-blotting of liver nuclear lysate with anti-GR antibody after immunoprecipitation with anti-ERα antibody; **i**) Calculation of bands intensity of GR normalized by ERα. Values are means ± SEM, ^*^P < 0.05, compared with control (*n* = 8). IB, Immuno-blotting; IP, immunoprecipitation

Hens fed betaine-supplemented diet showed significantly (*P <* 0.05) up-regulated mRNA expression of *VTGII* in the liver (Fig. 1a). Additionally, plasma level (Fig. 1b) and liver concentration (Fig. 1c) of VTG were also significantly higher (*P <* 0.05) in betaine-fed hens. Plasma level of estrogen (E2), which is well known for its role in the induction of hepatic *VTG*, did not differ between betaine-fed and control hens (Fig. 1d). *ERα*, but not *ERβ*, mRNA expression (Fig. 1e) was significantly increased, yet the protein content of ERα in the whole tissue lysate of liver did not differ (Fig. 1f).

### Hepatic GR expression and ERα/GR co-immunoprecipitation

Betaine-supplemented diet significantly (*P <* 0.05) up-regulated the hepatic expression of *GR* at both mRNA (Fig. 1g) and protein level (Fig. 1h). Co-immunoprecipitation of the liver nuclear lysates revealed direct protein/protein interaction between ERα and GR (Fig. 1j). Also, the content of ERα-associated GR tended to be higher (*P =* 0.07) in the liver of betaine-supplemented hens (Fig. 1i).

### Hepatic expression of the methyl transfer genes

**Fig. 2:**
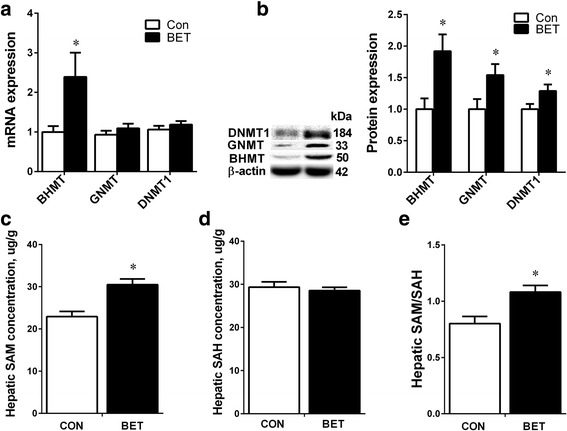
Effect of betaine supplemented-diet on Hepatic expression of the methyl transfer genes. **a**) mRNA expression of *BHMT*, *GNMT*, and *DNMT1*; **b**) Protein expression of BHMT, GNMT, and DNMT1; **c**) Hepatic SAM concentration; **d**) Hepatic SAH concentration **e**) SAM/ SAH ratio. Values are means ± SEM, ^*^P < 0.05, compared with control (n = 8). *BHMT*, betaine homocysteine methyltransferase; *GNMT*, glycine N-methyltransferase; *DNMT1*, DNA (cytosine-5-)-methyltransferase 1; SAM, S-adenosylmethionine; SAH, S-adenosylhomocysteine

Betaine significantly (*P <* 0.05) increased mRNA expression of *BHMT* (Fig. 2a), as well as protein content of BHMT, GNMT and DNMT1 (Fig. 2b) in the liver of laying hens. No significant alterations were detected for other genes involved in betaine metabolism and methyl transfer, which include *MAT2B*, *AHCYL1* and *DNMT3A* (Data not shown). S-adenosylmethionine (SAM), which is essential for DNA methylation, was significantly (*P <* 0.05) higher in the liver of betaine-treated hens (Fig. 2c). There was no change in the hepatic concentration of S-adenosylhomocysteine (SAH) in response to betaine feeding (Fig. 2d), yet SAM/SAH ratio was significantly (*P <* 0.05) higher in the liver of betaine-fed hens (Fig. 2e).

### Bisulfite sequencing analysis of *GR* promoter CpG methylation

**Fig. 3:**
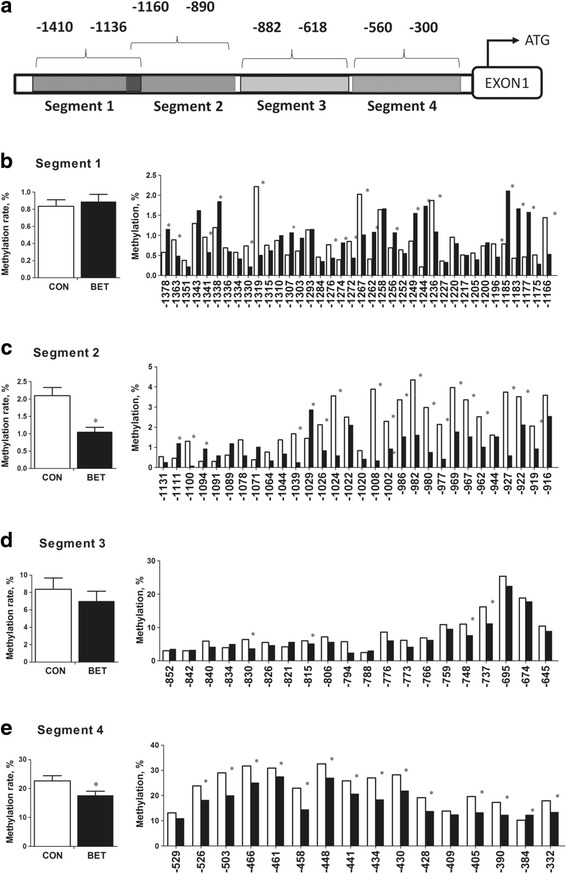
CpG methylation of GR gene promoter. **a**) Schematic diagram showing the promoter sequences of chicken *GR* gene (the 4 segments analyzed by BSP are highlighted 1–4); **b**-**e**) Average CpGs methylation level (left) and methylation status on each CpG site (right) in different segment. ^*^P < 0.05

BSP analysis for the genomic DNA revealed that feeding betaine modulated the methylation status of *GR* gene promoter in the liver of laying hens. In the present study, the methylation status of 4 segments upstream of the translation start codon (ATG) of the *GR* gene was studied (Fig. 3). The average methylation level and the methylation status on each CpG site are shown (Fig. 3b to Fig. 3e). Betaine significantly (*P <* 0.05) induced hypomethylation of most CpGs in Segment 2 located from −890 to −1160, and Segment 4 located from −300 to −560 upstream of *GR* translation start codon (ATG). The average methylation level of the other two segments was not significantly different between betaine and control groups.

## Discussion

In the present study, betaine supplementation did not affect the body weight of laying hens. This finding appears to contradict with some previous reports that dietary supplementation with betaine improves growth performance of broiler chickens [42, 43]. Nevertheless, the growth promoting effect of betaine is not consistent, some studies reported no effect of betaine on the body weight of chickens [44–46]. It seems that the effect of betaine on growth and body weight is dependent on the breed and the age of chickens, the dose and duration of betaine supplementation, and the composition of the basal diet. It is not suprising that feeding betaine for a relatively short period (4 wk in this study) did not greatly influence the body weight of hens at reproductive stage.

In the present study, laying hens fed betaine-supplemented diet had higher egg laying rate compared to their control counterparts, which is in line with other studies that reported increase of laying performance in betaine-treated chickens [29, 30, 47]. Moreover, we found that the increased laying rate was accompanied with increased hepatic concentration and plasma level of VTGII. Our results are consistent with previous reports about a positive correlation between VTG and egg production in chickens [5, 6] and killifish *(Fundulus heteroclitus)* [48].

Hepatic *VTG* expression is controlled by estrogen (E2) through ERα [4]. However, plasma E2 was not significantly changed by betaine treatment in this study. Although hepatic *ERα* mRNA level was higher in betaine group, the protein content of ERα did not differ in the liver whole cell lysates. Interestingly, hepatic expression of *GR* was significantly up-regulated at both mRNA and protein levels in betaine-supplemented hens. Furthermore, co-immunoprecipitation analysis revealed a direct protein-protein interaction between ER and GR, and the ERα-interacting GR tended to be higher in the liver nuclear lysate of betaine-supplemented hens. ER/GR interaction was first reported in an early study in which combined treatment of estradiol and glucocorticoid activated chicken *VTGII* transcription in vitro [49]. Similarly, estradiol alone was not able to induce significant levels of VTG synthesis and secretion in frog hepatocytes, while the presence of a glucocorticoid and thyroid hormone in addition to estradiol produced maximal induction [50]. In contrast to these studies, however, a study in rainbow trout demonstrated that dexamethasone caused a marked decrease in *ER* and *VTG* mRNA levels in the liver, and attributed such effect to possible inhibitory effects of GR [51]. It is noted that the plasma level of corticosterone was not altered (data not shown) in the present study. Therefore, the betaine-induced *GR* activation may be ligand-independent, in contrast with glucocorticoid-dependent *GR* activation [52].

In this study, dietary betaine supplementation enhanced hepatic methionine cycle, which is indicated by significantly increased protein content of BHMT, GNMT and DNMT1, as well as higher hepatic SAM concentration and SAM/SAH ratio. Our results support previous reports that betaine exposure increased hepatic SAM concentration and activated *GNMT* expression in piglets [53], and up-regulated hypothalamic *DNMT1* expression in chickens (28). DNMT1 is well known for its role in maintaining DNA methylation [54], and higher SAM/SAH ratio leads to global hypermethylation of the genome [55]. Interestingly, however, *GR* gene promoter was found to be hypomethylated in the liver of chickens fed betaine-supplemented diet. Although the lower promoter DNA methylation is in accordance with activated *GR* gene expression in this study, the mechanism by which betaine reduces the methylation status of *GR* promoter is unclear. Similar mismatches between enhanced methionine metabolism and lower methylation status of candidate gene promoters have been reported previously [56]. For instance, up-regulated *BHMT* and *AHCYL1* expression was associated with hypomethylation of phosphoenolpyruvate carboxykinase 2 and fructose-1,6-bisphosphatase 1 gene promoters in the liver of piglets prenatally exposed to betaine [40].

## Conclusions

Taken together, our findings indicate that betaine activates *GR* expression via hypomethylation of its promoter regions. Enhanced *GR* expression and GR/ERα interaction led to increased *VTGII* expression in the liver, which may contribute, at least partly, to improved egg production in betaine-supplemented laying hens. Our study is the first to report the hypomethylation-induced *GR* activation and a possible ligand-independent synergetic effect of GR/ERα interaction on the regulation of hepatic *VTGII* expression in the chicken. Certainly, further in-depth studies are required to show direct evidence of ERα/GR interaction and its action on chicken *VTG* gene transcription in response to betaine treatment.

## Abbreviations

BHMT: Betaine homocysteine methyltransferase
DNMT1: DNA (cytosine-5)-methyltransferase 1
E2: Estrogen
ER: Estrogen receptor
GNMT: Glycine N-methyltransferase
GR: Glucocorticoid receptor
SAH: S-adenosylhomocysteine.
SAM: S-adenosylmethionine
VTG: Vitellogenin
